# Nonalcoholic Fatty Liver Disease is Associated with Increased Carotid Intima-Media Thickness in Type 1 Diabetic Patients

**DOI:** 10.1038/srep26805

**Published:** 2016-05-26

**Authors:** Lei Zhang, Kaifeng Guo, Junxi Lu, Fangya Zhao, Haoyong Yu, Junfeng Han, Yuqian Bao, Haibing Chen, Weiping Jia

**Affiliations:** 1 Shanghai Diabetes Institute, Shanghai Key Laboratory of Diabetes Mellitus, Shanghai Clinical Center for Diabetes, Department of Endocrinology and Metabolism, Shanghai Jiaotong University Affiliated Sixth People’s Hospital, Shanghai 200233, China

## Abstract

A growing body of evidence suggests that NAFLD is associated with an increased risk of incident CVD events both in patients without diabetes and in those with type 2 diabetes (T2DM). However, no published data are available regarding the association between NAFLD and C-IMT in T1DM. A total of 722 patients (371 men) with T1DM were included in this cross-sectional study. The main outcome measures were detection of NAFLD, C-IMT and classical risk factors. The mean age of the subjects was 46.2 years, and 51.1% were male. The prevalence of NAFLD was 15.9%. NAFLD patients had a markedly greater C-IMT (0.81 ± 0.25 vs. 0.69 ± 0.18 mm; *p* < 0.001) and frequency of carotid plaque (28.9% vs. 16.9%; *p* < 0.05) than those without fatty liver. Moreover, the differences in C-IMT remained after adjusting for potential confounders. A stepwise linear regression analysis revealed that age (standardized β, 0.326; *p* < 0.001), NAFLD (standardized β, 0.151, *p* < 0.001), and hsCRP (standardized β, 0.115, *p* = 0.008) were independently associated with C-IMT in all subjects. Our data show NAFLD is associated with elevated C-IMT in T1DM independent of conventional cardiovascular disease risk factors.

Nonalcoholic fatty liver disease (NAFLD) has emerged as a growing public health problem worldwide. In the last decade, it has become apparent that the clinical burden of NAFLD is not restricted to liver-related morbidity or mortality, and the majority of deaths in NAFLD patients are related to cardiovascular disease (CVD) and cancer. These findings have fuelled concerns that NAFLD may be a new and added risk factor for extrahepatic diseases such as CVD. A growing body of evidence suggests that NAFLD is associated with an increased risk of incident CVD events both in patients without diabetes and in those with type 2 diabetes (T2DM)^1^,^2^,^3^. However, very few data are available regarding the prevalence of NAFLD and its association with the CVD in type 1 diabetes (T1DM).

Retrospective and prospective studies provide evidence of a strong association between NAFLD and the subclinical manifestations of atherosclerosis (increased intima-media thickness, endothelial dysfunction, arterial stiffness, impaired left ventricular function, and coronary calcification)^4^,^5^,^6^,^7^. Measurement of carotid intima-media thickness (C-IMT) and plaque burden by ultrasound is a well-validated and widely accepted screening approach to the prediction of CVD in asymptomatic subjects^8^. Importantly, the severity of the histological features of NAFLD appear to be independently correlated with increasing C-IMT^9^. Several cross-sectional studies^10^,^11^,^12^,^13^ have shown that NAFLD is strongly associated with increased C-IMT and increased coronary artery calcium scores in nondiabetic patients and in those with T2DM, although not all studies have agreed with these findings^14^,^15^. Moreover, studies have suggested that preclinical CVD (such as C-IMT, plaque, and endothelial dysfunction) are seen more frequently and to a greater extent in patients with T1DM, even at an early age. Some data suggest that its presence may predict CVD events. However, no published data are available regarding the association between NAFLD and C-IMT as a marker of subclinical atherosclerosis or subclinical CVD in T1DM.

It is important for both physicians and patients to be aware of increased CVD risk as a potential extrahepatic association of NAFLD in T1DM, as many of the tools used for screening are readily available in clinical practice (i.e., intravascular ultrasound or virtual histology). If disease is identified early, it should be managed appropriately with standard medical therapies. Therefore, the objective of this study was to investigate whether there is a difference in carotid atherosclerotic burden as a marker of subclinical CVD according to the presence of NAFLD in a large cohort of patients with T1DM and, if so, to evaluate whether NAFLD is related to C-IMT, independent of traditional CVD risk factors and metabolic syndrome.

## Research Design and Methods

### Study Participants

**Figure 1:**
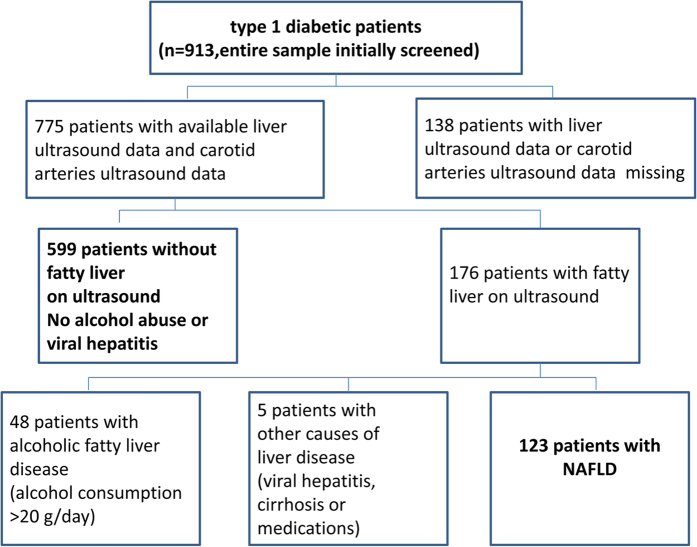
Details of the study design.

All patients diagnosed with type 1 diabetes (n = 913) who regularly attended Diabetic Clinic of Shanghai Jiao Tong University Affiliated Sixth People’s Hospital from January 2005 to December 2014 were initially eligible for the study. Type 1 diabetes was diagnosed by the typical onset of disease, the absolute dependence on insulin treatment for survival, and by the presence of severe deficiency of insulin secretion (i.e., very low or undetectable fasting C-peptide levels) and of anti-islet cell auto-antibodies. The following patients were excluded: (1) patients for whom a liver ultrasound examination or carotid arteries ultrasound data were not available (n = 138), (2) those who had known causes of chronic liver disease (alcohol-induced or drug-induced liver disease, Wilson’s disease, total parenteral nutrition, autoimmune or viral hepatitis) (*n* = 53). The remaining 722(79.1%) patients with type 1 diabetes were included in final analysis. Figure 1 shows the details of the study design. All participants were interviewed to obtain their history of alcohol consumption and smoking habits, and underwent an ultrasonographic evaluation of IMT, plaque in the carotid. The study was conducted in accordance with the Declaration of Helsinki and approved by the Ethics Committee of Shanghai Jiao Tong University Affiliated Sixth People’s Hospital. All study participants provided written informed consent prior to enrollment.

### Anthropometric and Biochemical measurements

Body weight was measured in light clothing and without shoes to the nearest half kilogram. Height, waist were measured to the nearest half centimeter. Body mass index (BMI) was calculated as the measured body weight in kilograms divided by the square of the measured height in meters. Blood pressure was measured using a standard mercury sphygmomanometer after the subject had been seated for at least 10 min.

Venous blood samples were drawn after an overnight fast. All the biochemical indices were measured on a Hitachi 7600 analyzer (Hitachi, Ltd., Tokyo, Japan). Fasting plasma glucose (FPG) was measured using the hexokinase method. Serum levels of total cholesterol (TC), triglyceride (TG), high-density lipoprotein cholesterol (HDL-C) and low-density lipoprotein cholesterol (LDL-C) were determined enzymatically. Alanine aminotransferase (ALT) and aspartate aminotransferase (AST) were measured using the UV method. GGT was measured by the Szasz-Persijn method. Hemoglobin A1c (HbA1c) was measured by high performance liquid chromatography with HLC-723G7 automated glycohemoglobin analyzer (Tosoh, Tokyo, Japan).

### Liver and carotid artery ultrasound

Guidelines for the diagnosis of nonalcoholic fatty liver diseases proposed by the Asia–Pacific Working Party were used to characterize subjects with NAFLD^16^. NAFLD was clinically defined as manifestations of B ultrasonography, ruling out the habit of drinking and the history of specific diseases that could result in fatty liver. Such diseases include viral hepatitis, drug-induced liver disease, Wilson’s disease, autoimmune hepatitis, and total parenteral nutrition. Abdominal ultrasonography was performed by experienced radiologists who were blinded to clinical presentation and laboratory findings. Hepatic steatosis was defined as a diffuse increase of fine echoes in the liver parenchyma compared with that in the kidney or spleen parenchyma based on standard criteria.

The common carotid arteries were scanned bilaterally using a high-resolution real-time B-mode ultrasonograph with an Acuson Sequoia 512 scanner (Siemens Medical Solutions, Mountain View, CA) equipped with a 5–13 MHz linear array transducer. Carotid ultrasonography was performed by three experienced ultra-sonographers under a standardised protocol. All subjects were examined in a supine position with their head turned 45 degrees from the site being scanned. Measurements were made manually on still images magnified to standard size on-line. The IMT was defined as the distance between the leading edge of the lumen-intima echo and the leading edge of the media-adventitia echo. The common carotid artery IMT was measured on-line in the posterior wall 10–20 mm proximal to the carotid bifurcation in a region free of focal plaque. Three measurements were made on each side, and the values were averaged to produce a mean IMT for each side. The mean Carotid intima-media thickness (C-IMT) was defined as the mean of the right and left IMT of the common carotid artery. A carotid plaque was defined as a distinct area of hyperechogenicity and/or protrusion into the lumen of the vessel with at least 50% greater thickness than the surrounding area.

Metabolic syndrome was diagnosed according to criteria proposed by the Adult Treatment Panel (ATP) III for Asian-Americans. In accordance with this definition, a person with type 1 diabetes was classified as having the metabolic syndrome if he/she had at least two of the following four components: (1) waist circumference > 90 cm in males or 80 cm in females; (2) triglycerides ≥ 1.7 mmol/L; (3) HDL cholesterol < 1.03 mmol/L in males and < 1.29 mmol/L in females or receiving lipid-lowering medications; and (4) blood pressure ≥ 130/85 mm Hg or receiving anti-hypertensive medications.

### Statistical Analysis

Data are means ± SD or frequencies. Normality was checked using the Kolmogorov-Smirnov test for continuous variables. If the data were distributed normally, independent sample t tests were used for comparisons of continuous variables between two groups. The Mann-Whitney U test was used if the data were not distributed normally, such as triglycerides, hsCRP, ALT, AST and GGT values, and the chi-squared test for categorical variables. For descriptive purposes, mean values were presented on skewed variables. Odds ratios (ORs) for Carotid plaque in each group were determined using logistic regression analysis, and the group without NAFLD was used as the reference. Multivariate-adjusted ORs are presented with 95% confidence intervals. An analysis of covariance was used to control for potential confounders for multivariate comparisons of C-IMT. Statistical correlations were determined by the nonparametric Spearman test. Stepwise liner regression analysis was used to elucidate the independent relationships between C-IMT and clinical parameters. The variables selected to enter into stepwise regression were those that correlated significantly with C-IMT(p < 0.05) by nonparametric Spearman test and variables that have a possible, confounding effect on the association between C-IMT and NAFLD (include smoking history and metabolic syndrome). All of the statistical analyses were performed by SPSS version 13.0 (IBM, Inc., Chicago, IL, USA). In all statistical test, *P values* < 0.05 was considered statistically significant.

## Results

### Clinical characteristics of the study population

The clinical characteristics of the study participants according to their NAFLD status are shown in Table 1. Overall, the 722 participants in the study had a mean age of 46.2 years, a mean body mass index (BMI) of 21.7 kg/m^2^, a mean HbA1c of 9.2%, and a mean duration of disease of 7.6 years. Of these subjects, 599 had negative liver ultrasound tests and the absence of excessive alcohol consumption (alcohol intake >20 g/day), viral hepatitis, or use of hepatotoxic medications, whereas the remaining 123 patients fulfilled the criteria for NAFLD. Of the 775 subjects initially included in the study (Fig. 1), NAFLD was present in 15.9% of the cohort.

Age, sex, HbA1c, diabetes duration, and fasting glucose did not differ between the two groups (without fatty liver and with NAFLD). Patients with NAFLD had significantly higher BMI, waist circumference, SBP, DBP, and a higher frequency of metabolic syndrome than those without NAFLD. They also had lower HDL-c and higher high-sensitive C-reactive protein (hsCRP), TC, TG, LDL-c, and liver enzyme levels, although the majority of NAFLD patients (i.e., 84.6%) had serum alanine aminotransferase (ALT) concentrations within the reference range. The proportions using antihypertensive (6.3% vs. 17.5%) or lipid-lowering (5.5% vs. 25.1%) medications were significantly higher in the NAFLD group.

### Comparison of C-IMT and carotid plaque between the diabetics with and without NAFLD

NAFLD patients had markedly greater C-IMT (0.81 ± 0.25 vs. 0.69 ± 0.18 mm; *p* < 0.001) than those without the condition, with no significant differences between males (0.73 ± 0.32; *n* = 371) and females (0.70 ± 0.16; *n* = 351), *p* = 0.117). Moreover, the frequency of carotid plaque was significant higher in NAFLD patients than in those without NAFLD (Table 1). An analysis of covariance (ANCOVA) was performed to verify whether the marked differences in C-IMT observed between subjects with and without NAFLD remained statistically significant after adjustment for potential confounders (Table 2). The differences remained unchanged after adjustment for age, sex, BMI, waist circumference, SBP, DBP, TC, TG, LDL-c, and HDL-c (model 1). Further adjustment for the presence of metabolic syndrome did not appreciably change the results (model 2). The results remained essentially unchanged even after additional adjustment for ALT, AST, GGT, hsCRP, and medication use (anti-hypertensives, and lipid-lowering agents). However, the relationship between NAFLD and the frequency of carotid plaque disappeared after adjusting for potential confounders (Table 2).

### Association between carotid intima-media thickness and clinical variables

Next, we investigated the relationships between C-IMT and a cluster of anthropometric parameters and biochemical indices (Supplementary Table 1). The analysis demonstrated significant positive associations of C-IMT with age (r = 0.531; *p* < 0.001), BMI (r = 0.226; *p* < 0.001), WC (r = 0.246; *p* < 0.001), diabetes duration (r = 0.240; *p* < 0.001), SBP (r = 0.280; *p* = 0.005), DBP (r = 0.164; *p* < 0.001), TC (r = 0.121; *p* = 0.002), LDL-c (r = 0.110; *p* = 0.004), GGT (r = 0.179; *p* < 0.001), and hsCRP (r = 0.361; *p* < 0.001) in the 722 subjects with T1DM, but no significant correlations were found between C-IMT and TG, fasting glucose, ALT, and AST (see Supplementary Table S1).

The independence of the association of C-IMT with NAFLD was also assessed by stepwise linear regression analysis in which we included all the study variables with significant correlations with C-IMT (including NAFLD) and a possible confounding effect on the association between C-IMT and NAFLD (including smoking history and metabolic syndrome) as covariates. According to the fully adjusted regression models, age (standardized β, 0.307; *p* < 0.001), NAFLD (standardized β, 0.148, *p* = 0.001), and hsCRP (standardized β, 0.148, *p* = 0.001) were independently associated with C-IMT in all subjects. Moreover, the association of NAFLD with C-IMT in subgroup analyses is shown in Table 3. The presence of NAFLD was significantly associated with C-IMT even when the study population was stratified to the median values of age, diabetes duration, BMI, SBP and HbA1c, and according to sex, presence/absence of the metabolic syndrome

### Association of hsCRP level with C-IMT and NAFLD

**Figure 2:**
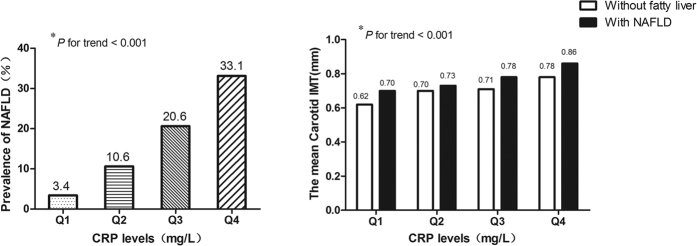
The prevalence of NAFLD and the mean Carotid IMT according to the hsCRP quartile categories. Q1, Q2, Q3, Q4 of hsCRP was <0.47 mg/L, 0.48–1.14 mg/L, 1.15–2.23 mg/L and >2.24 mg/L respectively. *p value for tr

As hsCRP level was significantly higher in patients with NAFLD than in those without fatty liver and as hsCRP was independently associated with C-IMT, we further analyzed the prevalence of NAFLD and the differences in mean carotid IMT after stratification according to hsCRP quartiles. The prevalence of NAFLD in subjects with hsCRP = Q1 was 3.4%. Notably, the rates increased to 10.6%, 20.6%, and 33.1% in subjects with Q2, Q3, and Q4 hsCRP levels, respectively (*p* < 0.001) (Fig. 2). Similarly, the mean carotid IMT increased gradually with increasing CRP in both subjects with NAFLD and in those without fatty liver (*p* < 0.001). Moreover, C-IMT was significantly higher in those with NAFLD than in those without fatty liver when CRP level was kept constant (Fig. 2).

## Discussion

Despite many studies of NAFLD, the prevalence, etiology, and consequences of the condition in conjunction with T1DM seem to have attracted relatively little attention. The prevalence of elevated liver enzymes has been reported to be higher in association with type 1 diabetes than in the general population^17^,^18^; NAFLD, as determined by ultrasound of the liver has also been considered very common in these patients^19^,^20^. More recently, in a longitudinal cohort of patients with type 1 diabetes, Harman *et al.* demonstrated that type 1 diabetes mellitus appears to be linked to a 1.875-fold increase in liver cirrhosis when compared with the general population in the UK^21^. Notably, liver-related outcomes among patients with Type 1 and Type 2 diabetes mellitus were similar in this study.

To our knowledge, this is the first study to investigate the association of NAFLD and carotid atherosclerotic burden in a large cohort of patients with T1DM. The major finding of this study was that NAFLD is strongly associated with C-IMT as an index of subclinical atherosclerosis^22^ in individuals with T1DM. Notably, these associations remained statistically significant even after controlling for several potential confounding factors, including a broad spectrum of classical CVD risk factors and metabolic syndrome. We also found that the frequency of carotid plaque was significantly higher in NAFLD patients than in those without NAFLD, although this relationship disappeared after adjusting for potential confounders.

Another novel observation of this study was that patients with NAFLD had significantly higher hsCRP levels than those without NAFLD, and the prevalence of NAFLD increased gradually with increasing CRP in T1DM. Moreover, we showed that hsCRP was independently associated with C-IMT in T1DM. This suggests that inflammatory factors (hsCRP) may play an important role in linking T1DM and NAFLD to subclinical atherosclerosis or CVD. However, the precise mechanism by which this is achieved remains unclear. In the general population, inflammation is a central pathological process of atherosclerosis^23^. Limited pathology data suggest that inflammation is more prominent in patients with diabetes than in nondiabetic control subjects^24^, and those with T1DM in particular are affected. C-reactive protein is elevated within the first year of diagnosis of T1DM^25^. The liver is a key metabolic organ and is central to the regulation of systemic inflammation. It is a generator as well as a target of various inflammatory and humoral factors, working in concert and against secreted molecules from adipose tissue, macrophages, and endothelial cells in the context of CVD initiation and progression^26^,^27^. The increasing severity of NAFLD likely represents a worsening of inflammatory and insulin-resistant states, with poorer cardiometabolic outcomes. hsCRP, which is primarily produced by the liver and is a marker of inflammation, has been linked to early or subclinical atherosclerosis^28^,^29^, and was shown to be an independent predictor of CV events in several large studies^30^,^31^. Our study confirmed this phenomenon and extends these findings to patients with NAFLD and T1DM. We showed that NAFLD was independently associated with C-IMT, independent of the classical cardiovascular risk factors in T1DM. We suspect that inflammatory factors (hsCRP) rather than other traditional risk factors may play an important role in this process. Further prospective and basic studies are required to confirm this suggestion.

The prevalence of NAFLD in T1DM was 15.9% in the present study (mean age, 46.2 years; mean BMI, 21.7 kg/m^2^; mean diabetes duration, 7.6 years). There is currently a lack of available information on the prevalence of NAFLD in T1DM, as only a few previous reports, all of which used ultrasonography to analyze the presence of liver fat, are available. Targher *et al.* observed fatty liver in 44.4% of a sample of 202 patients with T1DM (mean age, 43 years; mean BMI, 24.8 kg/m^2^; mean diabetes duration, 18.5 years)^19^. In another sample of 343 patients with T1DM, Targher *et al.* found a prevalence of 53.1% for hepatic steatosis (mean age, 44 years; mean BMI, 24.4 kg/m^2^; mean diabetes duration, 18 years)^32^. The differences in the prevalence of NAFLD between our findings and these two studies may relate to factors such as a higher BMI and an increased duration of diabetes in their samples, and the contribution of dietary pattern to the development of NAFLD may vary among different populations. Accordingly, our results may differ from those in other ethnic groups.

We found that serum ALT was within the normal range in 84.6% of subjects with T1DM. These results provide further evidence that a “normal” serum ALT level provides little diagnostic or prognostic value when assessing patients for NAFLD, as suggested by a previous study by Targher *et al.*^33^ indicating that approximately four fifths (~80%) of patients with T1DM and NAFLD had serum ALT levels within the reference range. Therefore, serum ALT levels or other liver enzymes appear to be insensitive markers for NAFLD^33^,^34^.

Our data showed that NAFLD is associated with elevated C-IMT independent of conventional cardiovascular disease risk factors in a large cohort of patients with T1DM. For patients with T2DM, however, the association between fatty liver and atherosclerosis is less clear, with conflicting results between various studies. Targher *et al.*^10^ studied a population of 200 diet-controlled type 2 diabetic subjects, reported that NAFLD patients had a markedly greater carotid IMT and that the increase of carotid IMT was largely explained by HOMA-estimated insulin resistance. In contrast, another two studies reported that hepatic steatosis was not associated with carotid atherosclerosis and suggested that the association of hepatic steatosis and cardiovascular disease might be just an epiphenomenon in diabetic population^14^,^15^. This inconsistency might be due to considerable heterogeneity in these studies in terms of outcomes measured as well as confounding variables not adequately adjusted for, and in the method of NAFLD diagnosis or the relatively small sample sizes. Furthermore, diabetes itself is considered a coronary-risk equivalent and so may have masked the association between NAFLD and carotid disease, especially when analysing relatively small sample sizes. Recently, Harman *et al.*^21^ demonstrated that longitudinal liver-related outcomes were similar comparing the T1DM cohort and respective type 2 diabetes cohorts—when adjusted for important confounder, moreover, the histological severity of NAFLD predicts C-IMT independent of traditional risk factors, insulin resistance, and metabolic syndrome components^9^. Taken together with our findings, we think that it is possible that NAFLD itself could, at least in part, contribute to accelerated atherogenesis in patients with both T1DM and T2DM, however, this need further basic research and prospective clinical studies to confirm in future.

Our findings may have important clinical and public health implications. These results support the suggestion that, among patients with T1DM, those with NAFLD should be considered at increased CVD risk. Therefore, the detection of NAFLD on a routine ultrasound examination should alert clinicians to the coexistence of multiple underlying CVD risk factors warranting evaluation and treatment as much as the risk of advancing liver disease. It should also be noted that it is currently unclear whether improving NAFLD will ultimately prevent the development and progression of CVD. Moreover, the prognostic value of NAFLD in CVD risk stratification remains debatable and will require further prospective studies and formal prediction analyses.

The present study had some important limitations. First, the cross-sectional study design limited our conclusions regarding the direction of the association or causal relationship between NAFLD and subclinical CVD risk. Our study showed that NAFLD is independently associated with increased C-IMT, but we did not assess cardiovascular events or mortality. Second, we did not perform liver biopsy with histological examination or magnetic resonance spectroscopy (MRS), and we used ultrasound to assess the presence of NAFLD. Liver biopsy is considered the gold standard for identifying steatosis. However, this invasive procedure is neither feasible nor ethical in a population-level study of this magnitude. Conversely, liver ultrasound is the most widely used non-invasive technique to detect fatty infiltration of the liver in clinical practice, and it has good sensitivity and specificity for detecting moderate and severe steatosis. Third, the contribution of dietary patterns to the development of NAFLD may vary among different populations. Accordingly, our results may differ from those in other ethnic groups. Finally, although liver ultrasound examination was available in the majority of patients who attended our diabetes clinic (i.e., 84.9% of the total sample) and there were no significant differences in demographic or main laboratory variables (including liver enzymes) between those who did and those who did not have a liver ultrasound examination, selection bias cannot be definitely excluded, and this may influence the prevalence of NAFLD in patients with T1DM more than its association with C-IMT.

In conclusion, NAFLD is associated with elevated C-IMT in T1DM, independent of conventional cardiovascular disease risk factors and metabolic syndrome. Moreover, hsCRP, an early and independent marker of subclinical atherosclerosis, may play an important role in linking TIDM and NAFLD to subclinical atherosclerosis or CVD.

## Additional Information

**How to cite this article**: Zhang, L. *et al.* Nonalcoholic Fatty Liver Disease is Associated with Increased Carotid Intima-Media Thickness in Type 1 Diabetic Patients. *Sci. Rep.* **6**, 26805; doi: 10.1038/srep26805 (2016).

## Supplementary Material

Supplementary Information

## Figures and Tables

**Table 1:** Characteristics of subjects according to NAFLD.

	Without fatty liver	With NAFLD	*p*-value
*n*	599	123	
Age (years)	46.0 ± 13.1	47.4 ± 13.2	0.437
Gender (%, male)	51.1	52.8	0.722
Diabetes duration (years)	7.71 ± 4.75	7.08 ± 3.41	0.450
BMI (kg/m^2^)	21.4 ± 3.7	23.5 ± 3.6	<0.001
Waist circumference (cm)	77.9 ± 9.5	85.0 ± 11.5	<0.001
SBP (mmHg)	122.1 ± 15.8	125.5 ± 13.9	0.009
DBP (mmHg)	74.9 ± 8.8	78.1 ± 8.7	0.002
HbA1c (%)	9.16 ± 2.57	9.48 ± 2.57	0.158
Fasting glucose (mmol/L)	8.48 ± 4.00	8.57 ± 3.85	0.814
TC (mmol/L)	4.59 ± 1.03	4.86 ± 1.32	0.014
TG (mmol/L)	0.93 ± 0.59	1.71 ± 1.80	<0.001
LDL-c (mmol/L)	2.67 ± 0.92	3.06 ± 1.13	<0.001
HDL-c (mmol/L)	1.44 ± 0.40	1.24 ± 0.39	<0.001
ALT (U/L)	19.82 ± 16.89	29.09 ± 20.46	<0.001
AST (U/L)	20.78 ± 13.34	25.83 ± 16.34	<0.001
GGT (U/L)	22.93 ± 42.94	29.85 ± 34.63	<0.001
hsCRP (mg/L)	1.34 ± 1.27	2.48 ± 1.62	<0.001
Elevated ALT^a^ (%)	5.7	15.4	<0.001
Metabolic syndrome (%)	34.4	55.3	<0.001
Lipid-lowering drug users	5.5	25.1	<0.001
Anti-hypertensive drug users (%)	6.3	17.5	0.009
Active smoker (%)	14.5	15.1	0.723
Carotid IMT (mm)	0.69 ± 0.18	0.81 ± 0.25	<0.001
Carotid plaque, %	16.9	28.9	0.005

Data are means ± SD or frequencies.

Abbreviations: BMI: Body mass index; HbA1c, hemoglobin A1c; SBP: Systolic blood pressure; DBP: Diastolic blood pressure; TC: total cholesterol; TG: triglyceride; HDL-c: high density lipoprotein cholesterol; LDL-c: low density lipoprotein cholesterol; ALT: Alanine aminotransferase; AST: Aspartate aminotransferase; GGT: γ-glutamyl transpeptidase, hsCRP, high sensitive C-reactive protein.

^a^Men > 50 U/L; women > 35 U/L.

**Table 2:** Association of mean C-IMT and frequency of carotid plaque with NAFLD.

	Without fatty liver	With NAFLD	*p*-value
*n*	599	123	
C-IMT			
Unadjusted	0.69 ± 0.18	0.81 ± 0.25	<0.001
Multiple adjusted association			
Model 1	0.700 ± 0.007	0.816 ± 0.018	<0.001
Model 2	0.701 ± 0.009	0.815 ± 0.011	<0.001
Model 3	0.704 ± 0.007	0.790 ± 0.019	<0.001
Carotid plaque, %			
Unadjusted	1	1.994 (1.218–3.264)	0.006
Multiple adjusted association			
Model 1	1	1.683 (0.819–3.459)	0.157
Model 2	1	1.670 (0.812–3.434)	0.163
Model 3	1	1.021 (0.450–2.317)	0.960

Data are expressed as mean ± SE and odds ratios ± 95% CI as assessed by an analysis of covariance or multivariate logistic regression analysis. The models adjusted as follows: model 1: Adjusted for age, sex, BMI, waist circumference, systolic blood pressure, diastolic blood pressure, TC, TG, LDL cholesterol, HDL cholesterol; model 2: as model 1, plus further adjustment for presence of the metabolic syndrome (as categorical variable); model 3: as model 2 plus ALT, AST, GGT, hsCRP, and medication use (anti-hypertensive, and lipid-lowering).

**Table 3:** Associations of ultrasound-diagnosed NAFLD with C-IMT in type 1 diabetic individuals stratified according to sex distribution, presence/absence of the metabolic syndrome or to the median values of age, diabetes duration, body mass index, systolic blood pressure, hemoglobin A1c and with insulin therapy alone.

Variable	β	95% CI	P value
Age < 49 years(n = 354)	0.357	0.103–0.202	<0.001
Age ≥ 49 years(n = 368)	0.245	0.074–0.180	<0.001
Men (n = 371)	0.104	0.004–0.124	0.036
Women (n = 351)	0.251	0.071–0.172	<0.001
Without the metabolic syndrome (n = 448)	0.199	0.076–0.208	<0.001
With the metabolic syndrome (n = 274)	0.141	0.011–0.110	0.016
Diabetes duration < 5 years (n = 356)	0.247	0.074–0.181	<0.001
Diabetes duration ≥ 5 years (n = 366)	0.122	0.012–0.125	0.018
BMI < 21.5 kg/m^2^ (n = 359)	0.196	0.065–0.211	<0.001
BMI ≥ 21.5 kg/m^2^ (n = 363)	0.143	0.021–0.121	0.005
HbA1c < 8.7 (n = 357)	0.264	0.083–0.194	<0.001
HbA1c ≥ 8.7 (n = 365)	0.276	0.089–0.201	<0.001
Systolic blood pressure < 120 (n = 336)	0.316	0.110–0.228	<0.001
Systolic blood pressure ≥ 120 (n = 386)	0.113	0.010–0.112	0.019

## References

[b1] Targher G., Day C. P. & Bonora E. Risk of cardiovascular disease in patients with nonalcoholic fatty liver disease. N Engl J Med. 363, 1341–50, 10.1056/NEJMra0912063 (2010). 20879883

[b2] Luo J., Xu L., Li J. & Zhao S. Nonalcoholic fatty liver disease as a potential risk factor of cardiovascular disease. Eur J Gastroenterol Hepatol. 27, 193–9, 10.1097/meg.0000000000000254 (2015). 25563143

[b3] Targher G. & Byrne C. D. Clinical Review: Nonalcoholic fatty liver disease: a novel cardiometabolic risk factor for type 2 diabetes and its complications. J Clin Endocrinol Metab. 98, 483–95, 10.1210/jc.2012-3093 (2013). 23293330

[b4] Oni E. T. *et al.* A systematic review: burden and severity of subclinical cardiovascular disease among those with nonalcoholic fatty liver; should we care? Atherosclerosis. 230, 258–67, 10.1016/j.atherosclerosis.2013.07.052 (2013). 24075754

[b5] Brea A. & Puzo J. Non-alcoholic fatty liver disease and cardiovascular risk. Int J Cardiol. 167, 1109–17, 10.1016/j.ijcard.2012.09.085 (2013). 23141876

[b6] VanWagner L. B. *et al.* Associations between nonalcoholic fatty liver disease and subclinical atherosclerosis in middle-aged adults: the Coronary Artery Risk Development in Young Adults Study. Atherosclerosis. 235, 599–605, 10.1016/j.atherosclerosis.2014.05.962 (2014). 24956534 PMC4124046

[b7] Kim D. *et al.* Nonalcoholic fatty liver disease is associated with coronary artery calcification. Hepatology. 56, 605–13, 10.1002/hep.25593 (2012). 22271511 PMC3830979

[b8] Lorenz M. W., Markus H. S., Bots M. L., Rosvall M. & Sitzer M. Prediction of clinical cardiovascular events with carotid intima-media thickness: a systematic review and meta-analysis. Circulation. 115, 459–67, 10.1161/circulationaha.106.628875 (2007). 17242284

[b9] Targher G. *et al.* Relations between carotid artery wall thickness and liver histology in subjects with nonalcoholic fatty liver disease. Diabetes Care. 29, 10.2337/dc06-0135 (2006). 16732016

[b10] Targher G. *et al.* Non-alcoholic fatty liver disease is associated with carotid artery wall thickness in diet-controlled type 2 diabetic patients. J Endocrinol Invest. 29, 55–60, 10.1007/bf03349177 (2006). 16553034

[b11] Sung K. C., Wild S. H., Kwag H. J. & Byrne C. D. Fatty liver, insulin resistance, and features of metabolic syndrome: relationships with coronary artery calcium in 10,153 people. Diabetes Care. 35, 2359–64, 10.2337/dc12-0515 (2012). 22829522 PMC3476919

[b12] Puig J. *et al.* Nonalcoholic fatty liver disease and age are strong indicators for atherosclerosis in morbid obesity. Clin Endocrinol (*Oxf*). 83, 180–6, 10.1111/cen.12698 (2015). 25510350

[b13] Kim K. S. *et al.* The association between non-alcoholic fatty liver disease and carotid atherosclerosis in subjects with within-reference range alanine aminotransferase levels. *Endocr J*. 60, 1295–301, doi: http://doi.org/10.1507/endocrj.EJ13-0269 (2013). 24047563 10.1507/endocrj.ej13-0269

[b14] McKimmie R. L. *et al.* Hepatic steatosis and subclinical cardiovascular disease in a cohort enriched for type 2 diabetes: the Diabetes Heart Study. Am J Gastroenterol. 103, 3029–35, 10.1111/j.1572-0241.2008.02188.x (2008). 18853970 PMC3638961

[b15] Petit J. M. *et al.* Nonalcoholic fatty liver is not associated with carotid intima-media thickness in type 2 diabetic patients. J Clin Endocrinol Metab. 94, 4103–6, 10.1210/jc.2009-0541 (2009). 19584186

[b16] Chitturi S. *et al.* Non-alcoholic fatty liver disease in the Asia-Pacific region: definitions and overview of proposed guidelines. J Gastroenterol Hepatol. 22, 778–87, 10.1111/j.1440-1746.2007.05001.x (2007). 17565630

[b17] West J. *et al.* Elevated serum alanine transaminase in patients with type 1 or type 2 diabetes mellitus. QJM. 99, 871–6, 10.1093/qjmed/hcl116 (2006). 17121768

[b18] Leeds J. S. *et al.* Abnormal liver function tests in patients with Type 1 diabetes mellitus: prevalence, clinical correlations and underlying pathologies. Diabet Med. 26, 1235–41, 10.1111/j.1464-5491.2009.02839.x (2009). 20002475

[b19] Targher G. *et al.* Prevalence of non-alcoholic fatty liver disease and its association with cardiovascular disease in patients with type 1 diabetes. J Hepatol. 53, 713–8, 10.1016/j.jhep.2010.04.030 (2010). 20619918

[b20] Targher G., Pichiri I., Zoppini G., Trombetta M. & Bonora E. Increased prevalence of cardiovascular disease in Type 1 diabetic patients with non-alcoholic fatty liver disease. J Endocrinol Invest. 35, 535–40, 10.3275/7875 (2012). 21795844

[b21] Harman D. J. *et al.* Prevalence and natural history of histologically proven chronic liver disease in a longitudinal cohort of patients with type 1 diabetes. Hepatology. 60, 158–68, 10.1002/hep.27098 (2014). 24585431

[b22] O’Leary D. H. & Polak J. F. Intima-media thickness: a tool for atherosclerosis imaging and event prediction. Am J Cardiol. 90, 18L–21L, 10.1016/S0002-9149(02)02957-0 (2002). 12459422

[b23] Libby P., Ridker P. M. & Hansson G. K. Inflammation in atherosclerosis: from pathophysiology to practice. J Am Coll Cardiol. 54, 2129–38, 10.1016/j.jacc.2009.09.009 (2009). 19942084 PMC2834169

[b24] Moreno P. R. *et al.* Coronary composition and macrophage infiltration in atherectomy specimens from patients with diabetes mellitus. Circulation. 102, 2180–4, 10.1161/01.CIR.102.18.2180 (2000). 11056089

[b25] Hayaishi-Okano R. *et al.* Elevated C-reactive protein associates with early-stage carotid atherosclerosis in young subjects with type 1 diabetes. Diabetes Care. 25, 1432–8, 10.2337/diacare.25.8.1432 (2002). 12145246

[b26] Wang Z. & Nakayama T. Inflammation, a link between obesity and cardiovascular disease. Mediators Inflamm. 2010, 535918, 10.1155/2010/535918 (2010). 20847813 PMC2929614

[b27] de Ferranti S. & Mozaffarian D. The perfect storm: obesity, adipocyte dysfunction, and metabolic consequences. Clin Chem. 54, 945–55, 10.1155/2010/535918 (2010). 18436717

[b28] Khera A. *et al.* Relationship between C-reactive protein and subclinical atherosclerosis: the Dallas Heart Study. Circulation. 113, 38–43, 10.1161/circulationaha.105.575241 (2006). 16380546

[b29] Thakore A. H. *et al.* Association of multiple inflammatory markers with carotid intimal medial thickness and stenosis (from the Framingham Heart Study). Am J Cardiol. 99, 1598–602, 10.1016/j.amjcard.2007.01.036 (2007). 17531588

[b30] Lavie C. J., Milani R. V., Verma A. & O’Keefe J. H. C-reactive protein and cardiovascular diseases–is it ready for primetime? Am J Med Sci. 338, 486–92, 486–492, 10.1097/MAJ.0b013e3181c61b66 (2009). 20010156

[b31] Pai J. K. *et al.* Inflammatory markers and the risk of coronary heart disease in men and women. *N Engl J Med*. 351, 2599–610, 10.1056/NEJMoa040967 (2004). 15602020

[b32] Targher G., Pichiri I., Zoppini G., Trombetta M. & Bonora E. Increased prevalence of chronic kidney disease in patients with Type 1 diabetes and non-alcoholic fatty liver. Diabet Med. 29, 220–6, 10.1111/j.1464-5491.2011.03427.x (2012). 21883436

[b33] Adams L. A., Angulo P. & Lindor K. D. Nonalcoholic fatty liver disease. CMAJ. 172, 899–905, 10.1503/cmaj.045232 (2005). 15795412 PMC554876

[b34] de Alwis N. M. & Day C. P. Non-alcoholic fatty liver disease: the mist gradually clears. J Hepatol. 48 Suppl 1, S104–12, 10.1016/j.jhep.2008.01.009 (2008). 18304679

