# Supplementary material for: Nonalcoholic Fatty Liver Disease is Associated with Increased Carotid Intima-Media Thickness in Type 1 Diabetic Patients
Source: Sci Rep. 2016 May 26;6:26805. doi: 10.1038/srep26805 (PMC4880892; doi:10.1038/srep26805)
Supplement: Supplementary Information [file srep26805-s1.doc]

**Nonalcoholic Fatty Liver Disease is Associated with Increased Carotid Intima-Media Thickness in Type 1 Diabetic Patients**

Lei Zhang*, Kaifeng Guo*, Junxi Lu, Fangya Zhao, Haoyong Yu, Junfeng Han, Yuqian Bao, Haibing Chen^▲^, Weiping Jia^▲^

Shanghai Diabetes Institute, Shanghai Key Laboratory of Diabetes Mellitus, Shanghai Clinical Center for Diabetes, Department of Endocrinology and Metabolism, Shanghai Jiaotong University Affiliated Sixth People’s Hospital, Shanghai 200233, China

**Word count:** 3523

**Number of figures and tables:** 5

Lei Zhang and Kaifeng Guo* contribted equally to this work.

Corresponding author and person to whom reprint requests should be addressed:

▲Haibing Chen, Ph.D

Department of Endocrinology and Metabolism, Shanghai Jiao Tong University Affiliated Sixth People’s Hospital, 600 Yishan Road, Shanghai 200233, China.

Phone: +86-21-64369181-58337

e-mail: [chenhb@sjtu.edu.cn](mailto:chenhb@sjtu.edu.cn)

▲Weiping Jia, Ph.D

Department of Endocrinology and Metabolism, Shanghai Jiao Tong University Affiliated Sixth People’s Hospital, 600 Yishan Road, Shanghai 200233, China.

Phone: +86-21-64369181-58922

e-mail: [wpjia@sjtu.edu.cn](mailto:wpjia@sjtu.edu.cn)

Lei Zhang,  stonez78@hotmail.com

Kaifeng Guo, guokaifeng7027@163.com

Junxi Lu, cissyludai@163.com

Fangya Zhao, xingfuquanli@yeah.net

Haoyong Yu, 13917766185@163.com

Junfeng Han tjhjf@163.com

Yuqian Bao, byq522@163.com

Haibing Chen, chenhb@sjtu.edu.cn

Weiping Jia, wpjia@sjtu.edu.cn

**Supplementary table S1.** Correlations of C-IMT with anthropometric parameters and biochemical indexes in 722 subjects with T1DM

| **Variable** | **r** | ***p*-value** |
| --- | --- | --- |
| Age | 0.531 | < 0.001 |
| BMI | 0.226 | < 0.001 |
| Waist circumference | 0.246 | < 0.001 |
| Diabetes duration | 0.240 | < 0.001 |
| Systolic blood pressure | 0.280 | < 0.001 |
| Diastolic blood pressure | 0.164 | < 0.001 |
| HbA1c | 0.085 | 0.034 |
| Fasting glucose | 0.008 | 0.825 |
| TC | 0.121 | 0.002 |
| TG | 0.058 | 0.131 |
| LDL-c | 0.110 | 0.004 |
| HDL-c | 0.021 | 0.583 |
| ALT | 0.038 | 0.321 |
| AST | 0.062 | 0.107 |
| GGT | 0.179 | < 0.001 |
| hsCRP | 0.361 | < 0.001 |

Abbreviations: BMI: Body mass index; HbA1c, hemoglobin A1c; TC: total cholesterol; TG: triglyceride; HDL-c: high density lipoprotein cholesterol; LDL-c: low density lipoprotein cholesterol; ALT: Alanine aminotransferase; AST: Aspartate aminotransferase; GGT: γ-glutamyl transpeptidase; hsCRP, high sensitive C-reactive protein.
